# Chronic migraine: nosographic and epidemiological issues

**DOI:** 10.1186/1129-2377-16-S1-A37

**Published:** 2015-09-28

**Authors:** Gian Camillo Manzoni, Paola Torelli

**Affiliations:** Department of Clinical and Experimental Medicine, University of Parma, Parma, Italy

The term “chronic migraine” (CM) was officially introduced in 2004 in the second edition of the International Classification of Headache Disorders (ICHD-2), which included it in the chapter on migraine at the three-digit level (code 1.5.1) among the complications of the disorder[1]. The latest edition of the classification (ICHD-3 beta version) published in 2013 still uses the term in the chapter on migraine but moves it to the two-digit level (code 1.3), after migraine without aura (1.1) and migraine with aura (1.2)[2]. According to the ICHD-3, a diagnosis of CM must be made when a patient who has been suffering from migraine for some time has had “headache occurring on 15 or more days per month for more than 3 months, which has the features of migraine headache on at least 8 days per month”.

The major drawbacks in the current systematization of this important chapter are the following: (a) the term used (CM) is ambiguous; and (b) the time pattern indicated in the diagnostic criteria is not adequate to define a homogenous case series of patients. In order to solve these drawbacks and be more adherent to the reality of clinical practice, CM as is currently known should be separated into two parts, depending on the severity of the headache[3]. One thing is having had headache on 15-20 days a month for 3-4 months. Quite another is having had headache each day of the month for several years. In the former case, we could use the term “high-frequency migraine”, including it at the three-digit level of migraine without aura. In the latter case, it would be better if we used the term “transformed migraine”, which is already well known in the literature and should be included at the three-digit level among the complications of migraine. This division of CM as we know it into two separate subgroups could be very helpful both in improving the clinical and healthcare management of patients and in providing much-needed availability of homogeneous case series for basic and pharmacology research.

Partly due to the nosographic ambiguities mentioned above, current epidemiological data are still scarce and rather conflicting. Based on the indications from the ICHD-2 and the ICHD-3, CM would have a 1-4% past-year prevalence rate in the general population, showing an even more marked predominance in women than does episodic migraine. More than half the CM cases would also have medication-overuse headache.
